# A theoretical model of the application of RF energy to the airway wall and its experimental validation

**DOI:** 10.1186/1475-925X-9-81

**Published:** 2010-11-27

**Authors:** Jerry Jarrard, Bill Wizeman, Robert H Brown, Wayne Mitzner

**Affiliations:** 1 Asthmatx, Inc, Sunnyvale, CA 94089 USA; 2 Johns Hopkins University, Baltimore, Maryland 20215 USA

## Abstract

**Background:**

Bronchial thermoplasty is a novel technique designed to reduce an airway's ability to contract by reducing the amount of airway smooth muscle through controlled heating of the airway wall. This method has been examined in animal models and as a treatment for asthma in human subjects. At the present time, there has been little research published about how radiofrequency (RF) energy and heat is transferred to the airways of the lung during bronchial thermoplasty procedures. In this manuscript we describe a computational, theoretical model of the delivery of RF energy to the airway wall.

**Methods:**

An electro-thermal finite-element-analysis model was designed to simulate the delivery of temperature controlled RF energy to airway walls of the in vivo lung. The model includes predictions of heat generation due to RF joule heating and transfer of heat within an airway wall due to thermal conduction. To implement the model, we use known physical characteristics and dimensions of the airway and lung tissues. The model predictions were tested with measurements of temperature, impedance, energy, and power in an experimental canine model.

**Results:**

Model predictions of electrode temperature, voltage, and current, along with tissue impedance and delivered energy were compared to experiment measurements and were within ± 5% of experimental averages taken over 157 sample activations.

The experimental results show remarkable agreement with the model predictions, and thus validate the use of this model to predict the heat generation and transfer within the airway wall following bronchial thermoplasty.

**Conclusions:**

The model also demonstrated the importance of evaporation as a loss term that affected both electrical measurements and heat distribution. The model predictions showed excellent agreement with the empirical results, and thus support using the model to develop the next generation of devices for bronchial thermoplasty. Our results suggest that comparing model results to RF generator electrical measurements may be a useful tool in the early evaluation of a model.

## Background

Recently a novel technique designed to reduce an airway's ability to contract by reducing the amount of airway smooth muscle (ASM) in the airway has been developed. The basic premise of the technique is related to the fact that ASM, when stimulated, causes airways to contract. Therefore, by reducing the amount of ASM in the airway wall this technique reduces the ability of the airway to contract. This technique has been examined in animal models [1,2] and as a treatment for asthma in human subjects [3-6]. This procedure, termed bronchial thermoplasty, involves the delivery of radio frequency (RF) energy directly to the airway wall. With this procedure a catheter is introduced into the lungs as illustrated in Additional file 1: Movie 1. The electrode array is expanded to contact the airway wall (a wire attached to the distal end of the electrode array expands the array when the wire is pulled proximally) and RF energy is applied to raise the temperature of the airway enough to cause destruction of the ASM but not damaging the surrounding tissues and subsequently reduce the ability of the airway to contract. This procedure has been shown to reduce the responsiveness of airways to methacholine chloride (Mch) challenge in dogs [2] and to alleviate symptoms and improve asthma control in patients with asthma [3-5]. Current data are promising and suggest a new treatment option for patients with asthma. The development of a next generation of devices used to administer bronchial thermoplasty could lead to more efficient delivery of energy and a reduction in overall procedure time. One step that may help in the development of the next generation of devices is a computational model capable of predicting the heat generation and transfer within the airway wall. Such a model will allow evaluation of next generation devices in-silico prior to animal and clinical validation thereby shortening development cycles and minimizing animal use.

In this manuscript we describe a computational, theoretical model of the delivery of RF energy to the airway wall. The model also includes prediction of the concurrent generation and transfer of heat within an airway wall. To implement the model, we use known physical characteristics and dimensions of the airway and lung tissues. In addition, the model incorporates a temperature control algorithm designed to simulate the temperature controlled delivery of RF energy using a six-electrode bipolar catheter design. While a number of groups have published work simulating and testing the delivery of RF energy to various biological tissues [7-16], none have applied such modeling to the unique anatomy in the mammalian lung.

To validate the model, its predictions were compared to empirical data obtained from experimental bronchoscopic RF energy delivery in the lungs of dogs.

## Methods

### A. Modeling of Airway Surrounding by Parenchyma

The model presented here was designed to simulate the delivery of temperature controlled RF energy to airway walls of the in vivo lung. In a living lung, we used four terms to deal with both the energy and heat delivery and heat loss. These are: 1) a term for the joule heating caused by the electrical current; 2) a term for the distribution of heating caused by thermal conductivity in the airway and parenchyma; 3) a term for the heat removal by the blood perfusion; and 4) a term for the heat removal by increased evaporation during the acute heating.

The model was constructed using Comsol Multiphysics, finite-element based analysis software, Version 3.5a (Comsol, Inc., Burlington, MA, USA). Coupled electrical and thermal equations were used to model the heat induced by radiofrequency (RF) current delivery to the airway wall and the transfer of this heat within the airway. The model includes a temperature feedback system which uses a PID (Proportional, Integral, Derivative) controller algorithm to simulate temperature controlled delivery of RF energy to the airway wall. The temperature is controlled to a target temperature that ramps up to 65°C over a period of 0.5 s and then remains constant at 65°C for an additional 2.5 s. This is the same target temperature that is employed in the RF generator used in our animal work and allows us to compare model output to RF generator measurements.

#### 1) Geometry and Mesh

**Figure 1:**
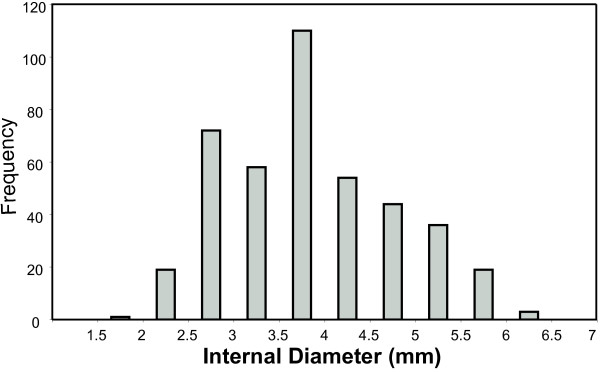
**Histogram of Airway Diameters Analyzed**.

Based on preliminary measurements in dogs, the diameters of treated airways ranged from 2 to 6.5 mm (see Figure 1) and were measured using a technique previously described [17]. One hundred eighty-eight airways were treated with an average diameter of 4.09 mm and a standard deviation of 0.94 mm. We used these anatomic data to physically scale the model to simulate the treatment of a 4 mm conducting airway using a six-electrode bipolar RF energy delivery catheter. This catheter consisted of an expandable electrode array with six equally spaced electrodes, where electrical energy flows between adjacent electrodes. The electrode dimensions and RF energy delivery scheme within the model were designed to mimic those used in previous animal studies so that the results of the animal work could be used to validate the model. Based on computed tomography (CT) measurements, airway wall thickness was assumed to be 10% of the internal airway diameter [18].

Although such CT measurements generally show increased wall thickness/diameter ratio as airways get smaller, some of this increase reflects a limited resolution of CT imaging that overestimates wall thickness and underestimates lumen diameter [19]. The wall thickness/diameter value also varies with lung inflation, but any chosen value for analysis could easily be changed to match actual anatomic measurements at different lung volumes or levels of airway contraction.

**Figure 2:**
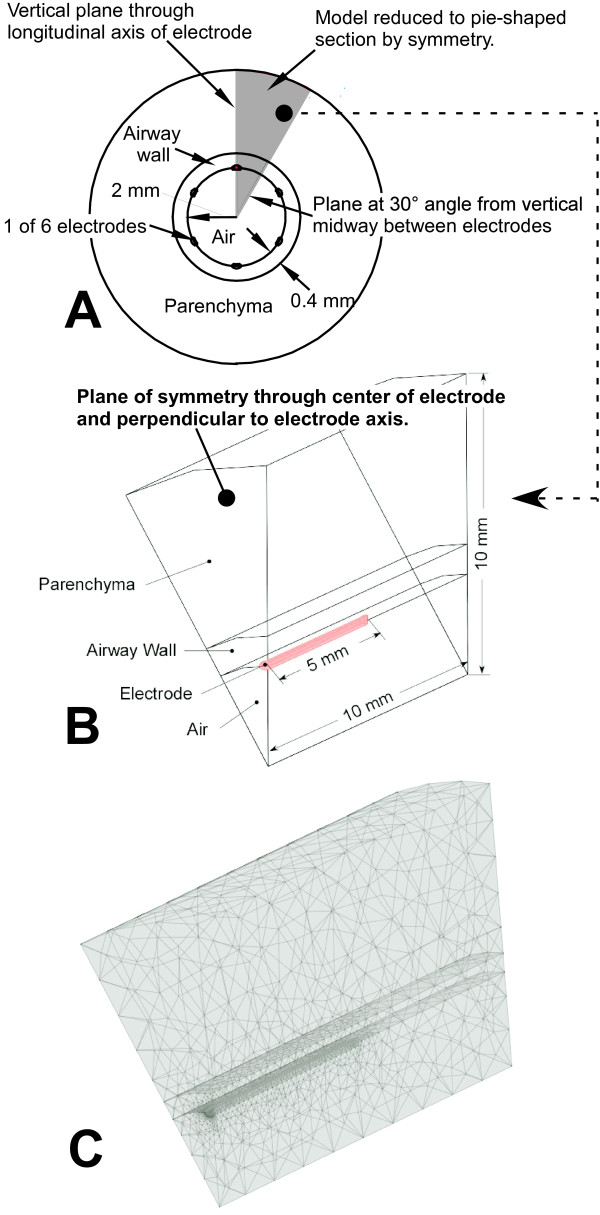
**Model Geometry and Mesh**. (A) Cross section of an airway illustrating the reduction of model size by symmetry. (B) Major dimensions of the model (C) Model mesh.

The model airway consists of an airway wall containing air within its lumen, and surrounded by parenchyma (see Figure 2). In current applications of bronchial thermoplasty [3-5], the device consists of an electrode array attached to a catheter which is introduced into the lungs through a bronchoscope. Once in place in an airway to be treated, the wires are deployed and bulge radially until light contact is made with the airway walls. Then RF energy is sent through the electrodes. In our model, the catheter electrodes were designed as D-shaped wires identical in width to the electrodes of the existing RF delivery catheter with the curved side of the wire partially embedded into the airway wall to simulate the way that the electrodes push into the tissue in-vivo. The electrodes were embedded in this way to simulate the manner in which the electrodes push into the airway mucosa during in-vivo treatments. Because the electrodes are evenly spaced around the inner surface of the airway, this circumferential symmetry allows a substantial reduction in the size of the model to 1/12 the size of the full model, Figure 2A, dramatically reducing the computation time. Symmetry along the centerline of the length of the electrode allowed us to reduce the size of the model by an additional 50% further reducing computation time, Figure 2B. The amount of parenchyma surrounding the airway was minimized to reduce computation time while maintaining the integrity of the model results in the region of interest near the electrode. In this region, the mesh (network of nodes at which precise calculations are made) was made very fine near the electrodes, and within the airway wall, and became coarser with radial distance. The mesh was thus refined sufficiently to ensure that the results were independent of the mesh. The mesh contained 22,668 elements. The boundary at the tissue-electrode interface was made up of 836 triangular elements with the longest dimension of any element being less than 0.5 microns. Figure 2B and 2C below shows the geometry and mesh of one slice of the symmetric model. The sensitivity to model radius and length of the average temperature within the airway wall, average temperature at the boundary between the airway and the parenchyma, and the heat flux across the airway/parenchyma boundary were checked and found to vary less than 1% when the model overall dimensions were varied by ± 50%. The bipolar current flow is concentrated within the airway wall between electrodes and this fact plus the high relative electrical and thermal conductivity of the airway wall, low electrical conductivity of the parenchyma, and short treatment time all combine to allow us to use a model with the overall dimensions shown in Figure 2B.

#### 2) Governing Equations

The RF generator used in the dog studies operated at a frequency of 480 kHz. At this frequency soft tissue is essentially resistive allowing us to use the DC electrical model in (Eq. 1). The temperature distribution within the tissue is governed by a modification of Pennes' bioheat equation (Eq. 2) [20]. The temperature dependence of tissue electrical conductivity was incorporated in the model and required that the equations be coupled.

The electrical field is found from solving Laplace's equation (Eq. 1)

(1) −∇⋅[σ(T)∇V]=0

Where ∇ is the gradient operator, V is the electric potential (volts), and σ (T) = σ_0_*EXP[0.015(T-T_0_)] is the temperature dependent conductivity (Siemens/meter) with σ_0 _= conductivity at normal body temperature (T_0 _= 37°C) and a temperature dependence of 1.5% per °C has been assumed [11] (see Berjano [21] for discussion on using exponential function to model temperature dependence of tissue conductivity).

The thermal model is a transient solution of the energy balance driven by the RF volume power density and includes joule heating in the tissue and electrode. (Eq. 2)

(2) $$\rho c\frac{dT}{dt}=\nabla \cdot (k\nabla T)+\sigma |\nabla V{|}^{2}-\rho_{b}c_{b}\omega (T-T_{body})-h_{fg}\cdot \frac{\partial m}{\partial t}$$

Where ρ = density (kg/m^3^); c = specific heat (J/kg-°C); T = temperature (°C); k = thermal conductivity (W/m-°C); σ = electrical conductivity (Siemens/meter); ρ_b _= density of blood (kg/m^3^), c_b _= specific heat of blood (J/kg·K), ω = the blood perfusion coefficient (s^-1^); T_body _= the ambient body temperature (37°C); h_fg _= latent heat of vaporization of water (J/kg); and ∂m = water mass volume density changing phase (kg/m^3^).

In Eq. 2, the term on the left of the equation represents the change in heat energy. The first term on the right is the change due to thermal conduction; the second, the change due to RF joule heating calculated using V and σ from Eq. 1; the third, heat removal due to blood perfusion; the last, heat removal due to evaporation in the parenchyma and along the airway inside surface.

##### Blood Perfusion

The method for estimating the removal of heat in a region of tissue by blood flow, the third term on the right hand side of eq. 2 above, was first proposed by Pennes in 1948 [22] and assumes that the temperature of the blood in capillaries equalizes with the temperature of the surrounding tissue and the heat removed depends upon the density and specific heat of the blood and well as the perfusion rate per unit volume of tissue (ω). To estimate **ω **we assumed that the cardiac output was uniformly distributed throughout the parenchyma and that parenchyma volume could be approximated by total lung volume (air and tissue). For the parenchyma a blood perfusion coefficient (ω) of 0.02 s^-1 ^was used based upon a cardiac output of 5.25 liters per minute for an average adult [23] and a lung volume of 4.3 liters [24]. It was also assumed that although human and canine cardiac output and lung volume are different the ratios are essentially the same. Pulmonary perfusion was assumed to be constant. It is unlikely to be changed by local spot heating in the airway and there is no experimental evidence available to suggest that it does.

For the smooth muscle in the airway wall the blood perfusion coefficient was assumed to be the same as for skeletal muscle and was estimated as 0.6 × 10^-3 ^s^-1 ^using total blood flow per minute to skeletal muscle at rest and the mass of skeletal muscle as a percentage of total body weight [23].

##### Evaporative Cooling

Evaporation is a type of vaporization that only occurs on the surface of a liquid, as compared to the generation of steam by boiling, which occurs throughout the entire mass of the liquid. Evaporation occurs when molecules at a liquid's surface has enough energy to break free. Molecules in water have a distribution of speeds, determined by their average kinetic energy, similar to the one described by the Maxwell distribution for gases[25]. At any given temperature there is a fraction of water molecules that have speeds well in excess of the average, and a few of these molecules have kinetic energies sufficient even to break the bonds holding them in the liquid. Evaporation increases with temperature and with decreasing humidity. Heat loss due to evaporation in airways has previously been modeled using finite-element-analysis [26]. We have extended our model to include evaporative heat loss in the parenchyma. The model assumes that evaporation occurs at the moist, spherical inner surface of an alveolus. The heat loss in a single alveolus due to evaporation was calculated using the equation below (Eq. 3). This equation was derived from a previously published equation for diffusive desiccation [27]. Heat loss per unit volume in the parenchyma was calculated by multiplying Eq. 3 by the total number of alveoli in the lung, assuming 300 million [28], and dividing by the total lung volume corresponding to a typical human (4.3 liters) [24]. Alveoli were assumed to be equally distributed throughout the parenchyma.

(3) $$h_{fg}\cdot \frac{\partial m}{\partial t}=h_{fg}\cdot 2\pi \cdot d\cdot M\cdot D_{H_{2}0}(T)\cdot (1-H_{r})\cdot C_{s}(T)$$

Where h_fg _is the latent heat of vaporization and is assumed constant at 2.4 × 10^6 ^J/kg, the value for water at 42°C [29]; d is the diameter of an alveolus assumed to be 0.3 × 10^-3 ^m [28]; M is the molecular weight of water, 0.018 kg/mole; DH_2_O = (0.171T + 20.84) × 10^-6 ^m^2^/s is the diffusion coefficient of water vapor; H_r _is relative humidity and assumed to be constant at 0.95; and C_s _= 0.2821 e^0.0588T ^is the saturation concentration of water vapor.Cs and DH2O were estimated from published data tables [27].

##### Temperature Control

Electrode temperature was controlled to 65°C for 3 seconds (including a 0.5 second ramp period) using the PID control equation shown below (Eq. 4), where the temperature error, e(t), is the difference between the electrode temperature and the target temperature; V_control _(t) is the voltage applied to the electrode; the first term on the right side of the equation is the error term multiplied by k_p _the proportional coefficient; the second term is the integral of the error multiplied by k_i _the integral coefficient and the last term is the derivative of the error multiplied by the derivative coefficient, k_d_, The values of the PID coefficients were selected to minimize the error between the electrode temperature and the target temperature and were set at k_p _= 20, k_i _= 60, and k_d _= 0.0. (Setting k_d _to zero in effect converted the controlled to a PI controller, but Eq, 4 was left unchanged in the model to allow the flexibility of including a derivative term in future models.) The shape of the target temperature curve was the same as that used in the animal study discussed above. The time steps for the transient analysis were left under solver control and were initially in the range of 1 × 10^-6 ^second initially and were increased into the 0.01 - 0.03 range after the simulation reached control temperature. The RF generator used in the dog study updated the control voltage every 50 ms.

(4) $$V_{control}(t)=K_{P}*e(t)+k_{i}\int_{0}^{t}e(\tau )d\tau +k_{d}\frac{de}{dt}$$

#### 3) Boundary Conditions

**Figure 3:**
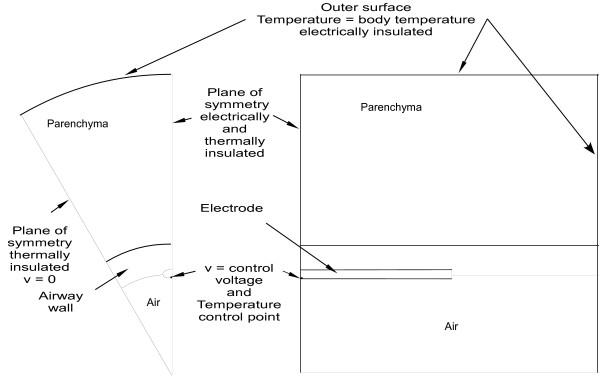
**Model Electrical and Thermal Boundary Conditions**.

A diagram showing the electrical and thermal boundary conditions for the model is shown in Figure 3 below. Electrically, the outer curved surface of the model, the plane of symmetry cutting through the center of the electrode along its longitudinal axis, and the plane of symmetry cutting through the center of the electrode perpendicular to the electrode axis are all electrically insulated. In the cases of the latter two boundaries, electrical insulation is appropriate since these are planes of symmetry (no current travels across these boundaries). The plane of symmetry rotated 30° from the electrode is grounded. The control voltage from Eq.4 is applied to the electrode cross-sectional area where the electrode is bisected by the plane of symmetry through the electrode's center. All internal boundaries are electrically continuous allowing current flow between sub-domains.

Thermally, the three planes of symmetry were insulated (adiabatic). The outer surfaces were constrained to body temperature. All internal boundaries are thermally continuous, allowing heat flow between sub-domains, except the air/airway wall boundary where evaporative cooling is assumed.

#### 4) Initial Conditions

All sub-domains and internal boundaries are set at zero volts initially. All tissue sub-domains are set to body temperature. The electrode and air are initially set at two degrees below body temperature [30].

#### 5) Material Properties

The electrical and thermal properties used in the model are given in Table 1 below. Electrical properties are for body temperature (37°C). Values for the electrical properties of tissues were calculated using the parametric model presented by Gabriel et al. [31]. Thermal properties of tissues are from Duck [32]. Properties not taken from Gabriel et al. or Duck are noted in parentheses in Table 1. The parenchyma was assumed to be 80% air/20% blood by volume and a weighted average used to estimate its properties. The airway wall was assumed to have the same properties as the trachea.

**Table 1:** Electrical and Thermal Properties of Materials Used in FEA Model

Material	σ (S/m)	k (W/m·K)	c (J/kg·K)	ρ **(kg/m**^**3**^**)**
Air	1e-16 [33]	0.030 [29]	1009 [29]	0.995 [29]
				
Blood	0.748	0.52	4176	1060
Trachea (airway wall)	0.359	0.5	3000	1500
Parenchyma	0.15	0.451[34]	1643	199
Stainless Steel 304	1.39e6 [35]	16.2 [35]	500 [35]	8030 [35]

### B. Experimental Testing of RF Energy Application in Dog Airways

All experiments were approved through the University of Utah's animal care and use committee. Three mongrel dogs weighting approximately 20 kg were initially anesthetized with propofol (approximately 6 mg/kg). Buprenex (0.02 mg/kg not to exceed 0.3 mg total dose) was administered as a subcutaneous injection. A 9 mm endotracheal tube was placed and secured.

The dogs were placed in dorsal recumbence on a recirculating warm water blanket and secured. A bronchoscope Y-adapter was attached to the endotracheal tube and connected to the anesthesia breathing circuit. Oxygen, room air and isoflurane were then administered, with the percentage of isoflurane adjusted to maintain a surgical level of anesthesia. Once the dog reached this level, the FiO_2 _was maintained at approximately 0.4.

Mechanical ventilation was initiated at approximately 15 cc tidal volume per kg of body weight, and respiratory rate was adjusted to maintain the oxygen saturation above 90%.

Airways within the lungs of dogs were treated using the 6-electrode bipolar catheter design as modeled above. The electrodes were 10 mm in length and were made of stainless steel wire having a rectangular cross-section The catheter contained (2) thermocouples on diametrically opposed electrodes which provided tissue temperature measurements to the RF generator to enable temperature controlled delivery of RF energy. The thermocouples were soldered to the electrodes and centered on the back, opposite the tissue contacting side of the electrodes A specially designed RF generator was used to deliver temperature controlled RF energy to the electrode array.

The treatment catheter was introduced into the lungs of the anesthetized dogs through the working channel of a standard 5 mm bronchoscope. The electrode array in the catheter was then expanded to contact the airway wall, and the RF generator activated, delivering RF energy controlled to 65 deg C to the airway wall for 3 seconds, including a 0.5 second ramp period. Immediately following an activation, the catheter was repositioned ≈10 mm from the prior treatment site for the next activation. This process of treating and repositioning the catheter was repeated to produce treatment of targeted airways with a baseline diameter range of 1.5 to 6.5 mm (see Figure 3).

Treatments were performed in the right lower lobe (RLL) for each of the three dogs. During all treatments, data from the RF generator were captured for later analysis. This data included measurements of voltage, current, power, impedance, and total energy.

## Results

A total of six parameters were plotted against time over a 3 second RF delivery period. These parameters were: measured control temperature, voltage, current, impedance, power, and energy. A total of 157 activations were completed in all dogs. All data are presented as average ± 1 standard deviation (n = 157). Since the results predicted by the model had no variation, the predicted values are plotted against time with no standard deviation.

**Figure 4:**
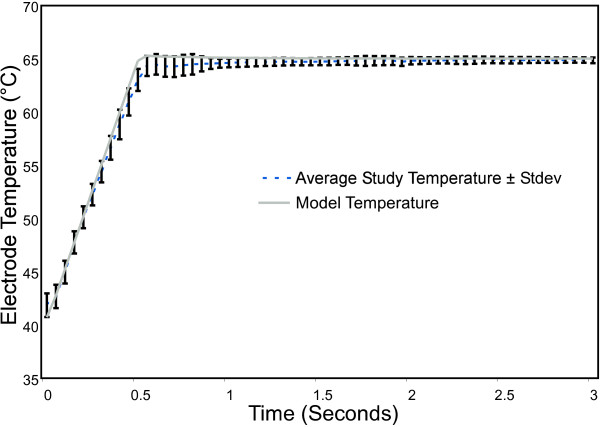
**Comparison of model controlled temperature at the center of the electrode to experimental data obtained from an in vivo canine model, n = 157**.

Figure 4 shows the time course of temperature measured at the inner surface of the airway wall during a 3-second activation. The temperature control algorithms within the model and in the RF generator demonstrated consistent results. They both raised the temperature of the tissue at the point of measurement to within approximately 1 degree C of the target temperature within 0.5 seconds and maintained the target temperature for the entire activation.

Thus the RF generator functioned as designed, with very little variation in temperature over the large range of airways treated. The slight delay in reaching the target temperature shown by the study temperature curve was probably the result of establishing the PID coefficients using an inflated excised dog lung on the bench, which did not have the energy losses of an in vivo lung, resulting in inadequate drive to consistently reach target temperature in 0.5 seconds.

The very slight difference in starting temperatures between experimental and model temperatures can be explained by two factors. (1) The body temperature from the animal study was slightly less than 40°C (A dog's normal body temperature is 38.9 ± 0.65°C [36]), while the model assumed a normal human body temperature of 37°C. (2) The research RF generator used in the animal study output a 0.1 A, 300 ms test pulse immediately prior to treatment and increased measured temperature by approximately 2°C at the end of the pre-treatment pulse, resulting in a starting temperature for the average experiment temperature of slightly less than 42°C. The model assumed the test pulse would increase temperature by 4°C resulting in a starting temperature of 41°C. The 4°C rise used in the model was based upon the temperature rise produced in a tissue phantom by a 0.1 A, 300 ms pulse. The tissue phantom (Jarrard & Associates, San Jose, CA) was made of a hydrogel having electrical and thermal properties similar to soft tissue such as cardiac muscle, smooth muscle, and liver. The actual average temperature rise in the study was lower due to the additional loses in the dogs not present in the tissue phantom. In addition, the pretreatment test pulse allowed for the collection of airway electrical parameters prior to the start of treatment.

**Figure 5:**
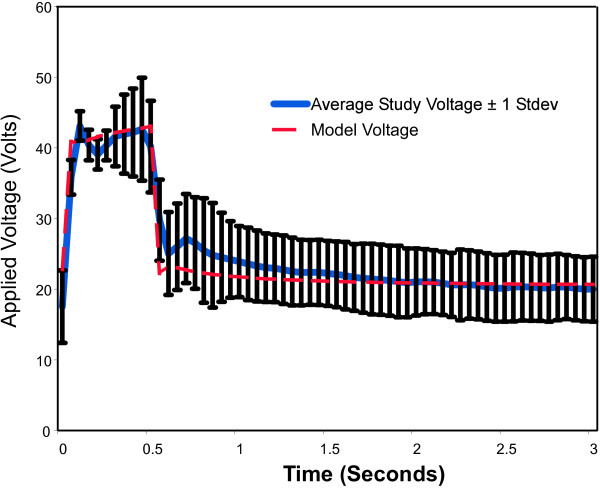
**Comparison of model applied electrode voltage to experimental data obtained from an in vivo canine model, n = 157**.

Plots following the time course of model results versus experimental data for voltage, current, impedance, power, and energy over a 3-second activation are shown in Figure 5 through 9. As can be seen, the model's predicted values agreed very well with the mean experimentally measured values during the 0.5 second ramp to control temperature and during the 2.5 second of steady state temperature control. The slightly higher than predicted experimental values during the 0.5 seconds after ramp-up were caused by the RF generator's slightly over damped temperature control algorithm. The local peak and slight oscillation in the measured voltage, current, and power curves at the start and end of the temperature ramp are due to a ringing in the output stage of the RF generator caused by the abrupt change in output voltage at the start and end of the temperature ramp.

**Figure 6:**
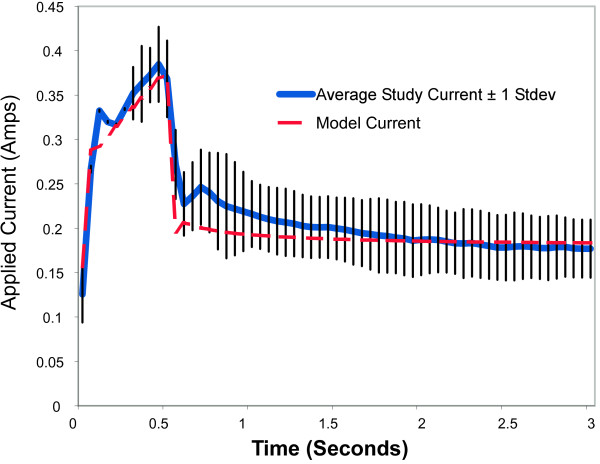
**Comparison of model total electrode current to average experimental data obtained from an in vivo canine model, n = 157**.

**Figure 7:**
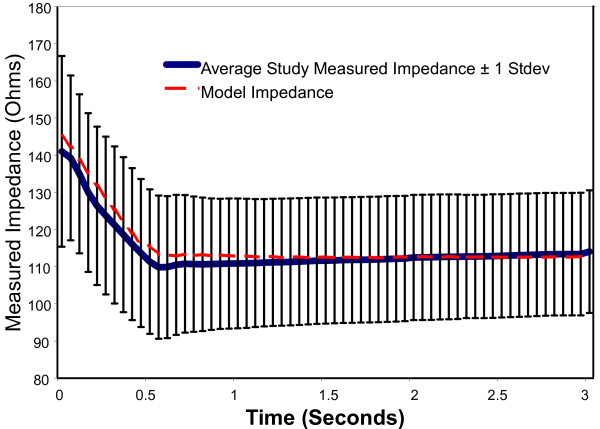
**Comparison of model electrode impedance to experimental data obtained from an in vivo canine model, n = 157**.

**Figure 8:**
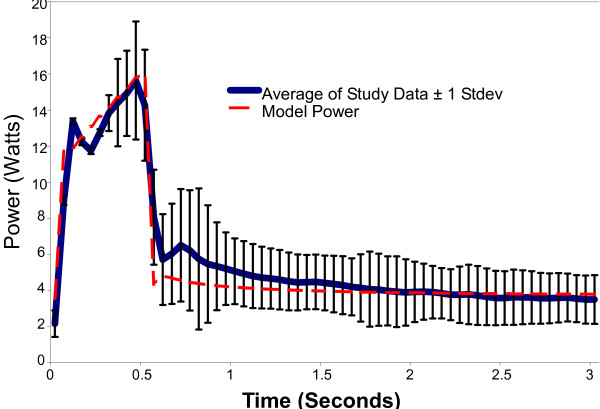
**Comparison of model delivered power to experimental data obtained from an in vivo canine model, n = 157**.

**Figure 9:**
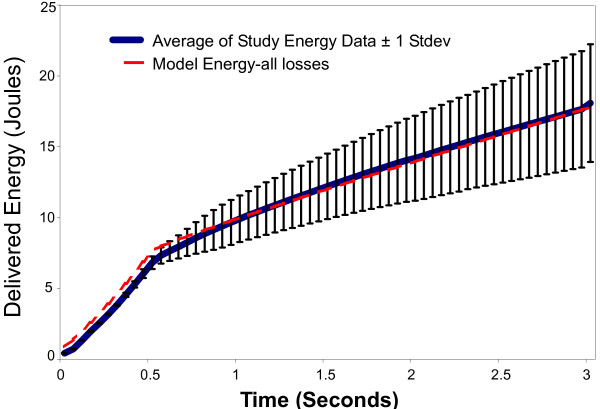
**Comparison of model delivered energy to experimental data obtained from an in vivo canine model n = 157**.

The difference between model and experimental starting energy in Figure 9 was caused by model assumptions regarding the pre-treatment test pulse discussed above. Since energy is the integration of power the model energy plot is shifted up by that amount.

## Discussion

Using a 3 D thermoelectric model, we were able to provide good predictions of how energy was transferred to the airway wall during a simulated application of temperature controlled RF energy to the airway wall using a six-electrode bipolar catheter. This model will be very useful to simulate different experimental conditions and greatly aid in the design of improved thermoplasty devices. Comparing a thermoelectric model to RF generator electrical output parameters is a new way of evaluating the performance of a model. To obtain a high degree of agreement between the model and experimental data required accurate estimates of both tissue electrical and thermal properties used in the model as well as model geometry. Alternate ways of evaluating an airway model, such as measuring temperatures at various sites in the treated tissue might yield more accurate data, but such measurements are not easily done in airways in vivo. Such an approach could be evaluated in phantoms, but unfortunately there are no good phantoms that match the heterogeneous tissue properties of airway in vivo, Furthermore, phantoms could not account for the evaporative heat loss and blood perfusion that occurs in vivo. Thus, our approach provides an excellent way of evaluating the efficacy of the application of RF energy under any specified experimental conditions.

The following discussion will first consider the new aspects of the modeling as applied to the lung, then will examine the testing of the model's predictions with in vivo data, and finally discuss the limitations of the approach with consideration of further advances.

### Model development and application

PID temperature feedback control was incorporated in the model because it was the control scheme used in the existing thermoplasty systems. The alternative would have been to empirically develop a voltage curve that produced the right temperature response at the temperature control point on the electrode, which would require several iterations [16]. An additional advantage is that the model is robust to variations in parameters such as airway diameter, wall thickness, tissue properties, etc. At most, changes in the model parameters will require changes to the PID coefficients. The alternative approach would require rebuilding the applied voltage waveform by trial and error for each change in the model parameters, but this is not likely to offer sufficient improvement to warrant the effort.

### Experimental validation of model

Figure 9 shows the time course of the energy delivered to the airway wall. The excellent agreement between the model and experimental results during the first 0.5 seconds was critically important, since it was during this time that the tissue was dynamically brought up to temperature. The model also tracks very well with experimental results during the constant temperature portion of the curve. These data thus provide strong support for the validity of the model's electrical and thermal parameters as well as the energy loss terms included in the model.

That evaporative heat loss was included in the model because of its physiological importance. Respiratory evaporation is known to be a significant factor in maintaining body temperature when ambient temperature approaches or exceeds normal body temperature in mammals such as dogs and sheep that do not have a significant number of sweat glands. For example, da Silva, et al. [37], showed that respiratory heat transfer in sheep at environmental temperatures above 30°C is minor and evaporative heat transfer through panting is the dominant factor in maintaining body temperature.

**Figure 10:**
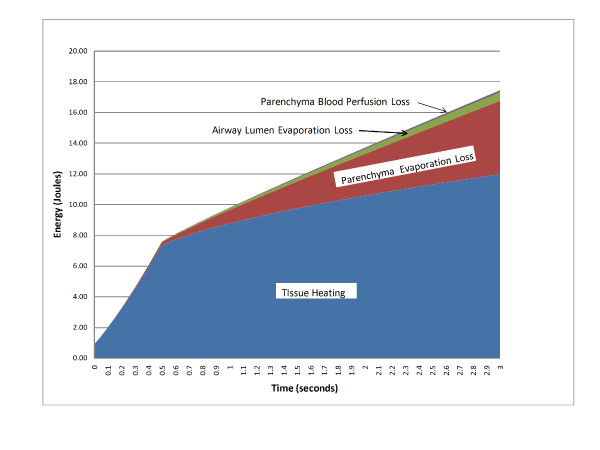
**Model Energy Budget**. Area chart showing breakdown of energy usage stacked to show contribution to total predicted energy delivered.

Incorporating evaporation heat loss was critical to model performance. The outer surface of the parenchyma was constrained to a body temperature of 37°C, and the initial air temperature was set at 35°C [30]. Figure 10 is an area chart of the energy uses predicted by the model, with the energies stacked to give the predicted total energy delivered each treatment. As would be expected, most of the energy goes to the therapeutic heating of tissue (includes heat flow from one area to another). The other major uses of energy are (1) heat loss due to evaporation within the parenchyma, (2) inner airway heat loss due to evaporation, and (3) heat loss due to parenchymal blood perfusion. Heat loss due to blood perfusion within the airway wall and heat loss due to convection loss within the airway were insignificant and not included in the chart.

Losses due to airway wall blood perfusion by the bronchial circulation were insignificant, which is not surprising, since the bronchial circulation is normally on the order of 1% of the total pulmonary blood flow that perfuses the surrounding parenchyma (where heat loss due to perfusion was significant). Convective cooling at the inner surface of the airway was assumed insignificant since air at the treatment site is near body temperature and there is reduced airflow due to blockage by the treatment catheter and the bronchoscope.

Most of the heat loss was due to water evaporation, particularly in the parenchyma. In the initial formulations of the model, we had not included evaporative losses, but this absence resulted in an almost flat energy curve along the constant temperature portion of the curve. The high heat of vaporization of water plus the fact that the alveolar surface area in a normal lung is very large led us to suspect that evaporative heat loss could play a large role in contributing to the overall heat loss, and this was proven by the improved fit when evaporative cooling was included.

The ratio of alveolar surface area to lung volume for an average adult human male was used in the equation to estimate evaporative loss in the parenchyma. The close agreement between model results and canine data indicates that the ratio is also good for dogs and possibly for other mammals. Since evaporation in the parenchyma is the major loss term in the model we checked the sensitivity to alveolar surface to lung volume ratio by varying it ± 20% in the model and looked at the changes in energy delivered versus baseline. The energy required to reach control temperature (energy at 0.5 seconds) remained at 7.7 joules. The total energy only varied ± 2.8% (± 0.5 Joules) from baseline. We did not check the sensitivity of luminal evaporation but would expect changes from baseline to be insignificant since it is based upon a modification of the equation used for parenchyma evaporation.

Figure 9 shows the time course of the changes in airway wall electrical impedance upon activation. As steady-state temperature was approached, the impedance was expected to gradually decrease, and this was predicted by the model. This decrease in impedance resulted from the temperature dependent nature of electrical conductivity. The small, gradual increase in impedance observed in the experimental data is unexplained and no attempt to address this small effect was included in the model.

Although we present data for this model analyzed for a 4 mm airway with a 0.4 mm wall thickness, it is straightforward to input other airway sizes to make the predictions more general. However, the model is quite robust in its stability to even moderate changes. For example, if we run a simulation with a wall thickness of 15% and a diameter 50% bigger and smaller than what was presented here, these changes result in only minor differences, such that the new calculated electrical parameters are within the error bars of the experimental data.

### Future model developments

The ultimate purpose of this model is twofold. First, from a basic physiologic perspective, it is useful to be able to take known anatomical and physical properties and use them to quantify electrical and energy transmission in the three dimensional airway tree. Second, the model can be used help design a system that shortens treatment time while producing the most effective extent of airway smooth muscle reduction. To that end it would be advantageous to add a method of predicting tissue damage of the various airway wall components and surrounding tissue. However, at present we have not implemented such additions to the model, because data on the differential electrical and thermal properties of the different tissues in the lung are still being acquired. Nevertheless, a more detailed model may be useful to implement sensitivity analyses to assess which components and which properties might be most important to include.

## Conclusions

In this study we implemented a novel computational model of an airway wall embedded in lung parenchyma that allowed analysis of the energy distribution following the delivery of RF current through discrete electrodes contacting the luminal surface of the airway. This model used reasonable assumptions of what was known about the physical properties of lung tissues. The model incorporated the same control scheme as used in currently existing RF delivery devices (i.e., a temperature feedback PID controller), and this allowed for comparison of model predictions with experimental RF generator electrical measurements. This approach eliminated the need for possibly problematic measurements using implanted temperature sensors. The model also demonstrated the importance of evaporation as a loss term that affected both electrical measurements and heat distribution. The model predictions showed remarkable agreement with the empirical results, and thus support the potential ability to use the model to develop the next generation of devices for bronchial thermoplasty.

## Competing interests

Jerry Jarrard and Bill Wizeman are full time employees of Asthmatx, inc. and hold stock ownership or options with Asthmatx (stock options are not currently traded).

## Authors' contributions

All authors have read and approved the final manuscript.

## Supplementary Material

Additional file 1 **Animation of bronchial thermoplasty catheter use**. A bronchoscope being positioned in a distal airway followed by the deployment of the radio frequency (RF) electrode array and the thermal treatment of three contiguous sections of the airway segment. (Animation courtesy of Asthmatx, Inc.) Click here for file
